# Value of C-Reactive Protein as a Risk Factor for Acute Coronary Syndrome: A Comparison with Apolipoprotein Concentrations and Lipid Profile

**DOI:** 10.1155/2012/419804

**Published:** 2012-10-16

**Authors:** Magdalena Krintus, Marek Kozinski, Anna Stefanska, Marcin Sawicki, Karolina Obonska, Tomasz Fabiszak, Jacek Kubica, Grazyna Sypniewska

**Affiliations:** ^1^Department of Laboratory Medicine, Ludwik Rydygier Collegium Medicum, Nicolaus Copernicus University, 9 Sklodowskiej-Curie Street, 85-094 Bydgoszcz, Poland; ^2^Department of Cardiology and Internal Medicine, Ludwik Rydygier Collegium Medicum, Nicolaus Copernicus University, 9 Sklodowskiej-Curie Street, 85-094 Bydgoszcz, Poland

## Abstract

*Objective*. To investigate whether assessment of C-reactive protein (CRP) and apolipoproteins, besides the traditional lipid profile, enhances the assessment process for the risk of acute coronary syndrome (ACS). *Methods*. The study group consisted of 220 consecutive patients admitted to hospital within the first 6 hours from the onset of chest pain. Patients were diagnosed with unstable angina (*n* = 96), non-ST-elevation myocardial infarction (NSTEMI; *n* = 57), or ST-elevation myocardial infarction (STEMI; *n* = 67). ACS patients were compared with 116 healthy volunteers in a case-control study. The serum was assayed on admission for CRP, apolipoproteins ApoAI and ApoB100, and lipid parameters. *Results*. The highest concentrations of CRP were found in NSTEMI and STEMI, with a median value four-fold higher in ACS patients than in controls (*P* < 0.0001). Only CRP significantly increased the probability of ACS development (adjusted odds ratio for a 1 mg/L increase 1.90; 95% confidence interval [CI] 1.34–2.89) and explained 90% of the variation for ACS development. Similarly, we demonstrated the highest diagnostic accuracy for CRP among all investigated markers (area under the curve 0.80; 95% CI 0.75–0.85). *Conclusions*. Our study indicates that CRP superiorly to apolipoproteins and lipid profile facilitates the risk stratification for ACS occurrence.

## 1. Introduction

Despite great progress in pharmacotherapy and interventional treatment, acute coronary syndromes (ACS) remain the major cause of mortality and morbidity in the modern world [1]. Inflammation plays a key role in the initiation and promotion of atherosclerotic lesions and can trigger ACS by the induction of plaque instability. C-reactive (CRP) protein is an extensively studied inflammatory factor whose prognostic value in cardiovascular diseases in recent years has become increasingly important [2–7]. Additionally, CRP is no longer merely considered a marker but also emerges as a mediator of atherosclerosis [8, 9].

Thus, considering the generally available lipid profile a tool for risk assessment, it seems that CRP and the lipid profile besides the patient's clinical characteristics could lead to the most tangible benefit for assessing the risk of ACS development. On the other hand, in recent years the value of CRP in this setting has been extensively debated. In a large meta-analysis of 22 prospective studies in healthy individuals, Danesh et al. found that in studies published after 2000, the prognostic value of CRP in predicting ACS was much weaker than in a meta-analysis of research results from before 2000 [10]. This led to a critical review of the role of CRP in the assessment of occurrence of cardiovascular events and less optimistic application of its determination for the primary and secondary prevention [10, 11]. In contrast with these data, a recent individual participant meta-analysis of 160,309 patients confirmed substantial continuous associations between CRP concentration and the risks of ACS, ischaemic stroke, and vascular mortality [2].

One of the best known risk factors and a primary target of therapy is elevated concentration of low-density lipoprotein cholesterol (LDL-C). Despite many studies indicating the need for determination of LDL-C in patients at risk of ACS, it is more and more often suggested that focusing solely on LDL-C is not an optimal diagnostic and therapeutic strategy [12]. This attitude is driven by several important limitations associated with LDL-C. Triglyceride-rich lipoproteins, including very low-density lipoproteins (VLDL) and intermediate-density lipoproteins (IDL), also exert atherogenic properties. Patients, who achieved a reduction in LDL-C even below 70 mg/dL, still are at the so-called residual risk of atherothrombotic events [13]. Atherogenic factors that influence this residual risk consist of elevated IDL and VLDL levels and the presence of small dense LDL particles, which are not detected in a basic lipid panel. 

The coexistence of high triglycerides (TG) and low high-density lipoprotein cholesterol (HDL-C), called atherogenic dyslipidemia, is associated with elevated apolipoprotein B concentration. While the role of apolipoproteins in coronary risk assessment is an evolving debate, the apoB : apoA-I ratio is becoming increasingly important [14, 15]. The results of the AMORIS and INTERHEART studies demonstrated that the apoB : apoA-I ratio was the strongest predictor for myocardial infarction among all investigated variables and most importantly, like CRP, was able to identify subjects at high risk even when LDL-C values were considered normal [16, 17].

As the routine determination of apolipoproteins and CRP in ACS risk stratification remains controversial, this study aims to investigate whether evaluation of these markers enhances, besides the traditional lipid profile, the assessment process for the risk of ACS development.

## 2. Material and Methods 

### 2.1. Study Design and Conduction

The study was designed as a case-control study. We screened consecutive patients admitted due to the initial diagnosis of ACS to the Department of Cardiology and Internal Medicine at The University Hospital in Bydgoszcz within the first 6 hours from the onset of chest pain. The exclusion criteria were as follows: (i) chronic heart failure (New York Heart Association class II–IV), (ii) acute heart failure (Killip class II–IV), (iii) pulmonary embolism within 6 months preceding the enrolment, (iv) creatinine concentration >176.8 mmol/l, (v) ACS within 6 weeks preceding the enrolment, (vi) the presence of features suggestive of an active inflammatory process on admission, and (vii) therapy with steroids, immunosuppressive agents, and nonsteroidal anti-inflammatory drugs (excluding low doses of aspirin). 

Among 267 subjects, who fulfilled the requirements of the study according to the inclusion and exclusion criteria, 47 patients were diagnosed with unspecified chest pain or other heart diseases and were excluded from further analysis. The final study group consisted of 220 patients (91 females and 129 males, aged 64 ± 12 years). All these patients met clinical criteria of ACS. Electrocardiographic examination was performed on admission and thereafter if clinically indicated. Echocardiography, stress tests, and cardiac catheterization were performed if needed. Patients with ACS were subsequently definitely diagnosed with unstable angina (UA; *n* = 96), non-ST-elevation myocardial infarction (NSTEMI; *n* = 57), or ST-elevation myocardial infarction (STEMI; *n* = 67). Clinically healthy volunteers (61 women and 55 men, aged 52 ± 9 years) with no evidence of present renal, metabolic or inflammatory disease, heart failure, and recent myocardial infarction served as controls. 

Dyslipidemia was defined by at least one abnormal level of serum lipid parameters: total cholesterol (TC) >5.2 mmol/L, TG > 1.7 mmol/L, LDL-C > 2.6 mmol/L, HDL-C < 1.3 mmol/L for women and <1.0 mmol/L for men according to the modified definition of Third Report of the National Cholesterol Education Program [18]. Hypertension was diagnosed if systolic blood pressure exceeded 140 mmHg and/or diastolic blood pressure was above 90 mmHg. Baseline characteristics of study participants are presented in Table 1.

The study protocol was approved by the Bioethics Committee at Nicolaus Copernicus University in Torun Collegium Medicum in Bydgoszcz and written informed consent was obtained from all patients and controls.

### 2.2. Blood Sampling and Laboratory Analyses

Venous blood samples were collected from patients on hospital admission within 6 hours of chest pain onset. Fasting venous blood samples from controls were collected in the morning. Serum was assayed on admission for cardiac troponin I (cTnI) and lipid parameters (ARCHITECT ci8200, Abbott Diagnostics). Any increase of cTnI above 0.032 ng/mL (the 99th percentile for the healthy population measured with a 10% coefficient of variation) was considered a positive result.

High-sensitivity CRP (hsCRP) was measured using the BN II System nephelometer (N High-Sensitivity CRP; Siemens Healthcare Diagnostics, Deerfield, IL, USA), providing excellent precision with the coefficient of variation reported by the manufacturer of less than 10%. Coefficients of variation for hsCRP estimated in our laboratory were below 3.5% and below 4.5% for hsCRP concentrations below 1 mg/L and above 3 mg/L, respectively. The lower limit of CRP detection was 0.17 mg/L. Serum apoA-I and apoB100 concentrations were measured in samples stored frozen at −80°C no longer than 6 months (ARCHITECT ci8200) and the ratio of apoB : apoA-I was calculated. ApoB concentration >0.9 g/L was classified as abnormal. TC : HDL-C <4 and TG : HDL-C <3 were regarded as optimal. According to Walldius and Jungner [16] the apoB : apoA-I ratio over 0.8 for women and 0.9 for men was considered to represent high risk ofACS.

### 2.3. Statistical Analysis

We performed an internal pilot study for estimating the final sample size. CRP concentrations for the first 100 patients in the study group and for the first 50 patients in the control group were, respectively, 5.55 ± 7.19 and 1.41 ± 1.61 mg/L. Based on these results and assuming a 2-sided alpha value of 0.05, we calculated that enrolment of 220 patients in the study group and 116 patients in the control group would provide a 99.8 power to demonstrate a significant difference in CRP concentrations between patients with and without ACS. We decided to obtain such high power to be able to conduct credible multivariate analyses. 

The Kolmogorov-Smirnov test was used to assess normality of distribution of investigated parameters. Data were expressed as mean ± standard deviation and median with 25th–75th percentiles. Variances in the two groups with normal distribution were examined using the Levene's test. Comparison between the groups was performed by using the Chi-square test for categorical variables, the unpaired Student's *t*-test, ANOVA followed by the Tukey test for normally distributed variables, and the Mann-Whitney *U*-test and the Kruskal-Wallis test for nonnormally distributed variables, with the post hoc Dunn's tests. Pearson correlation was used to analyze associations between variables. Variables with nonparametric distribution underwent logarithmic transformation. *P* value <0.05 was considered statistically significant. Logistic regression was performed to determine associations between baseline parameters and the risk of ACS development. Clinical utility of laboratory parameters was determined by analysis of Receiver Operating Characteristic (ROC) curves. Statistical analysis and sample size calculation were performed using SPSS 17.0 software package (SPSS, Chicago, IL, USA) and Statistica 10.0 for Windows (StatSoft, Tulsa, OK, USA).

## 3. Results

### 3.1. Characteristics of Participants and Major Findings of Basic Statistics

Baseline characteristics of the study participants, including lipid parameters, CRP, and apolipoproteins concentrations constituting major cardiovascular risk factors, are shown in Table 1. All quantitative variables differed significantly between both groups. Patients with ACS, compared with controls, had higher concentrations of TC, LDL-C, non-HDL-C, and TG, but lower levels of HDL-C and apoA-I. They also presented significantly higher values of atherogenic indexes such as TC : HDL-C, LDL-C : HDL-C, apoB : apoA-I, and TG : HDL-C, reflecting insulin resistance, as well as fourfold higher median concentration of C-reactive protein in comparison with the control group. Both groups were characterized by the presence of dyslipidemia, hypertension, and smoking. A quarter of ACS patients were diagnosed with type 2 diabetes.

In the course of further analysis the patients were divided into groups based on the specific clinical diagnosis (Table 2). UA patients formed the largest group, also characterized by lower age compared with those with STEMI and NSTEMI. Statistically significant differences between the UA and STEMI groups were found for LDL-C, non-HDL-C and the LDL-C : HDL-C, ratio. ApoB concentration and the apoB : apoA-I ratio were substantially higher in STEMI patients compared with the UA and NSTEMI groups, while between the latter two no major differences were found. CRP concentration was markedly higher in patients with STEMI and NSTEMI compared with those with UA.

Correlation analysis showed no significant relationships between serum CRP and other variables in the control group. In contrast, in the ACS group, weak but statistically significant correlations were observed between CRP concentration and the apoB : apoA-I ratio (*R* = 0.16;  *P* = 0.02), the TG : HDL-C ratio (*R* = 0.15;  *P* = 0.02), the LDL-C : HDL-C ratio (*R* = 0.15;  *P* = 0.02), and the TC : HDL-C ratio (*R* = 0.14;  *P* = 0.03), respectively. Also a weak but statistically significant negative correlation between CRP and HDL-C (*R* = −0.14;  *P* = 0.04) was found in this group.

### 3.2. The Probability of ACS Presented Using Logistic Regression

The odds ratio for the occurrence of ACS among the conventional risk factors was highest for hypertension, then followed by age, dyslipidemia, and smoking (Table 3). The probability of ACS occurrence, depending on the measured laboratory parameters and calculated ratios, was presented using logistic regression after adjustment for age, gender, and smoking status successively. In order to avoid data redundancy, the variables have been divided into primary variables (Table 4) and secondary variables (data not presented). Measured analytes have been identified as the primary variables, while variables resulting from calculations of other parameters were classified as secondary. The estimator of risk for ACS occurrence was the logistic odds ratio, which was given along with a 95% confidence interval and significance level.

The results of logistic regression for the primary variables are presented in Table 4. The first model designed, Model 0 unadjusted, was highly significant and based on the result of a pseudomeasure of quality of the fit-*R* ^2^ Nagelkerke explained 47% of the variation for ACS occurrence. In this model, all TC, HDL-C, LDL-C, TG, apoA-I, and apoB did not facilitate the risk stratification for ACS development. Only CRP significantly increased the probability of the occurrence of ACS (by 60%). Model 1 after adjustment for age was also highly significant and explained approximately 74% of the variation for the presence of ACS. Among the primary variables only CRP increased the risk of ACS (by approximately 55%). In contrast, apoA-I significantly reduced the probability of ACS occurrence. Another model, which was also highly significant and explained approximately 75% of the variability for ACS development, was designed after adjustment for age and sex. TC affected the risk of ACS in 12%, whereas HDL-C and apoA-I markedly lowered this risk. In this model, CRP still remained the strongest predictor of ACS occurrence. The last of the models, model 3 for the primary variables, was adjusted for age, sex, and smoking status. This model was also highly significant and explained approximately 86% of the variation for the presence of ACS. CRP increased the probability of ACS occurrence roughly by 66%.

Table 5 displays the results of logistic regression models with both the primary and secondary variables. The analyzed parameters were naturally correlated with one another. Size distortions associated with the collinearity of independent quantitative variables depended on the value of the correlation coefficient between the two variables. A preliminary analysis of correlations between lipid variables from Table 5 revealed only moderate strength of the correlation coefficients between these variables. In addition, we run a tolerance analysis that indicated that these variables can be tolerated in the model.

Model 0, adjusted neither for age, sex, nor smoking, was highly significant and based on the result of a pseudo measure of quality of the fit-*R* ^2^ Nagelkerke explained 49% of the variation for the presence of ACS. In this model, CRP was the only significant predictor for the occurrence of ACS, increasing the probability of disease by 60%. Model 1, after adjustment for age, was highly significant and explained 75% of variation for ACS development. In this model, CRP was the only variable enhancing the probability of ACS. While the predictive value of CRP alone in Model 1 was 56%, adjustment for age and sex, as performed in Model 2, increased it to 64%. The highest odds ratio for ACS occurrence was obtained in Model 3 (including adjustment for age, sex, and smoking status) for the primary and secondary variables. This model was also highly significant and explained 90% of variation for the presence of ACS. In this model, elevated CRP concentration nearly doubled the likelihood of ACS.

### 3.3. Diagnostic Accuracies of Investigated Markers

**Figure 1:**
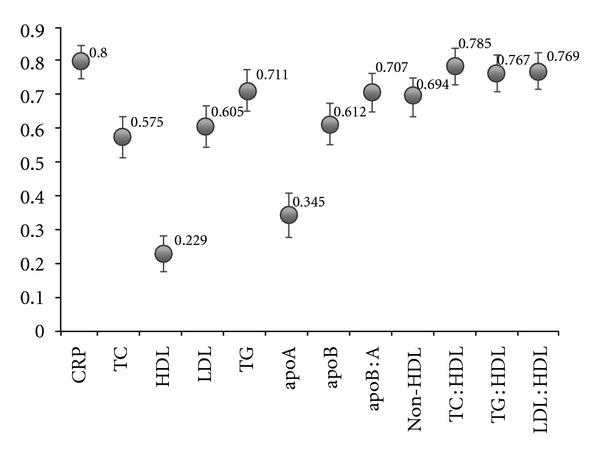
The diagnostic value of measured and calculated variables for the occurrence of acute coronary syndrome. apoA: apolipoprotein A-I; apoB: apolipoprotein B; apoB : A: apolipoprotein B to apolipoprotein A-I ratio; CRP: C-reactive protein; HDL: high-density lipoprotein cholesterol; LDL: low-density lipoprotein cholesterol; LDL : HDL: low-density lipoprotein cholesterol to high-density lipoprotein cholesterol ratio; nonHDL: non-high-density lipoprotein cholesterol; TC: total cholesterol; TC : HDL: total cholesterol to high-density lipoprotein cholesterol ratio, TG: triglycerides; TG : HDL: triglycerides to high-density lipoprotein cholesterol ratio.

Finally, we evaluated the ROC curves to assess diagnostic accuracies of investigated variable for the prediction of ACS occurrence. The highest level of discrimination of ACS was found for CRP (area under the curve [AUC] = 0.80), however, this was not significantly different from the diagnostic accuracy of three other variables: TC : HDL-C (AUC = 0.78), LDL-C : HDL-C, and TG : HDL-C (AUC = 0.77). The diagnostic accuracies of all variables are shown in Figure 1. 

For all measured parameters sensitivity, specificity, positive predictive value, and negative predictive value were calculated. Among them, the most valuable results were obtained for C-reactive protein and lipid ratios (Table 6). A ROC analysis revealed an optimal cut-off point for CRP of 0.85 mg/L.

## 4. Discussion 

Our study clearly indicates that CRP possesses a higher prognostic value in terms of ACS prediction than apolipoproteins and lipid profile. ACS cases showed 4-fold higher median CRP concentration on admission than healthy controls. The difference in CRP level between patients with and without ACS was much more pronounced in our data than for apolipoproteins and lipid parameters and remained significant after adjustment for age, sex, and smoking status. Furthermore, assessment of the diagnostic accuracy confirmed a very good ability of CRP to discriminate between cases and controls. To increase the robustness of our findings we enrolled into our study only patients diagnosed with ACS within first 6 hours from the onset of chest pain. We established this time frame restriction in the inclusion criteria to minimize a potential impact of necrosis-related inflammatory reaction on CRP concentration.

Elevated CRP concentration in patients who presented to hospital with chest pain due to ACS was previously demonstrated by others [19, 20]. In our study the highest CRP levels were observed in NSTEMI and STEMI patients and were considerably higher than in UA patients. These observations are consistent with results of other researchers showing higher CRP concentrations in patients with myocardial infarction than with stable or unstable coronary artery disease [19–22]. Additionally, some investigators have reported CRP levels to be higher in patients with STEMI than in those with NSTEMI and noted their further and significant decrease in patients with UA [21, 23]. However, in these trials blood sampling beyond 6 hours from the onset of chest pain was in agreement with the study protocols. Therefore, the observed variation in CRP concentrations among the types of ACS might be at least partially attributed to the differences in the area of the infarcted myocardium. On the other hand, there are also reports demonstrating lack of significant differences in CRP concentrations at baseline among patients with ACS [24, 25]. It should be also acknowledged that elevated CRP concentration on admission is suggested to be a marker for anatomic complexity of culprit lesions [23, 24]. 

CRP concentration above 3 mg/L, close to this present in our ACS patients, is currently recommended by the Centers for Disease Control and Prevention (CDC) and the American Heart Association (AHA) as an independent predictor of cardiovascular events in patients at intermediate global risk (Class of recommendation IIa, Level of Evidence B) [6]. Nevertheless, the optimal cut-off point for hsCRP test determined by ROC analysis in our study was 0.85 mg/L. This may indicate a higher diagnostic value of the test for low concentrations of CRP. Our cut-off point is close to 1 mg/L, being a lower limit for the intermediate risk category according the CDC and AHA statement. Calculations performed using our data for the cut-off point of 3 mg/L revealed a slightly lower sensitivity but higher specificity than for the cut-off point of 0.85 mg/L. In line with our findings, the results of the JUPITER trial support lower than 3 mg/L cut-off point for increased cardiovascular risk. In this randomized, placebo-controlled study of 17,802 apparently healthy persons with LDL-C concentration below 2.6 mmol/L but with hsCRP level of 2.0 mg/L or higher, rosuvastatin significantly reduced the incidence of major cardiovascular events [26]. Interestingly, another recent study has addressed the prognostic efficacy of hsCRP in ACS patients presenting within 6 hours from the onset of chest pain and identification of the optimal cut-off value to determine the long-term prognosis [27]. In this cohort hsCRP level above 1.1 mg/L had the optimal positive and negative predictive values. 

Our study group, diagnosed with ACS, was diverse in terms of established risk factors including dyslipidemia, hypertension, diabetes, and smoking; each of them is capable of explaining the occurrence of high CRP concentrations. ACS patients have a higher risk of subsequent cardiovascular events, featuring myocardial infarction, stroke, and death. Primary and major strategies to diminish premature cardiovascular morbidity and mortality should include the identification and treatment of established risk factors, especially hypertension, dyslipidemia, smoking, obesity, and diabetes. Although the study group was characterized by dyslipidemia, the median concentrations of lipids, except for LDL-C and non-HDL-C, were within normal limits. Also, the median concentration of apolipoprotein A-I and apoB remained normal. The calculated atherogenicity indexes, except for TC : HDL-C and LDL-C : HDL-C, also did not exceed the reference values. However, although remaining within normal ranges, there were statistically significant differences concerning all parameters, compared with the control group. It might be explained by the lower prevalence of risk factors in the control group, since as evidenced by the baseline characteristics, the prevalence of dyslipidemia, hypertension, and smoking in this group was significantly lower and there were no patients with diabetes. The reduced values of the lipid parameters could be also due to statin therapy, which in the study group was received by 69% of patients prior to admission. Despite ongoing statin therapy the residual cardiovascular risk can still be significant in patients with dyslipidemia [28]. In our study, significant correlations between CRP and atherogenic indexes were observed only among those diagnosed with ACS. As we know, these indicators demonstrate the atherogenicity and the presence of small dense LDL as well as insulin resistance [29].

There is growing evidence that targeting other lipids, such as triglycerides and HDL-C, is an important way to reduce the residual cardiovascular risk, particularly in patients with frank metabolic dysfunction [30]. In our study we observed that out of the basic lipids and apolipoproteins only HDL-C and apoA-I importantly limited the risk of cardiovascular events, but not in every model, and that after switching to models incorporating the secondary variables such as atherogenicity indexes, this effect was negligible. In contrast, we found that CRP significantly improved risk prediction models' accuracy and that it was the strongest predictor of ACS in each of the logistic regression models designed. ACS risk was highest for CRP compared with the lipid parameters, atherogenicity indexes, and apolipoproteins (odds ratio = 1.9) in the model designed after adjustment for age, sex, and smoking. To the best of our knowledge, despite the large number of publications on CRP, to date only few studies have been published concerning the clinical utility of CRP in comparison with apolipoproteins and simple lipid indexes calculated on the basis of the routine lipid profile.

Based on a subanalysis of the PROVE IT-TIMI 22 trial including ACS patients receiving statin therapy, Ray et al. concluded that the addition of hsCRP to lipid-based measurements significantly improved risk prediction, while apoB : apoA-I, TC : HDL-C, non-HDL-C and LDL-C provided a similar risk prediction accuracy [31]. In contrast, the INTERHEART case-control study suggested that apoB : apoA-I provides the highest odds ratio for myocardial infarction occurrence compared with LDL-C and TC : HDL-C, [32]. In the present study, CRP significantly improved risk prediction irrespective of lipids, apolipoproteins, and their calculated ratios included. Moreover, based on ROC curve analysis, CRP becomes the most important discriminator of ACS cases compared with other parameters. Besides the role of CRP, considering the discrimination power assessed by ROC analysis, also TC : HDL-C, LDL-C : HDL-C, and TG : HDL-C showed similar and significant performance for ACS prediction. The discrimination power was assessed quantitatively as the AUC, which was 0.80 for CRP, 0.78 for TC : HDL-C and 0.77 for LDL-C : HDL-C, and TG : HDL-C. In contrast, other studies showed lower discriminative usefulness of CRP. The AUCs for CRP in the NPHS-II trial and in the EAS trial were 0.61 (95% confidence interval 0.57–0.66) and 0.62 (95% confidence interval 0.57–0.67), respectively [33]. However, in these studies the measurement of CRP at baseline was performed in healthy individuals and perhaps due to this fact it provided only limited discrimination for cardiovascular events compared with the population burdened with additional risk factors. 

In our study we failed to demonstrate any relation of apoB and apoB: apoA-I with the onset of the cardiovascular event. The lack of influence of apolipoproteins and the lipid ratios on the occurrence of ACS could be explained by the earlier use of statins in approximately 70% of patients; however, it is not consistent with the fact that statins also decrease CRP level independently of lowering LDL-C and apoB concentrations [34]. This leads to the suggestion of the pivotal role of inflammation in comparison to the subsidiary involvement of small dense atherogenic lipoproteins in the process leading to ACS. 

Finally, it remains an unsolved issue whether CRP directly contributes to atherothrombotic events and may be a potential therapeutic target, or if it just reflects an increased risk for unfavourable outcome as a bystander marker [8, 9]. A large body of basic scientific evidence suggests that CRP possesses proatherogenic features. As demonstrated by Williams et al., CRP increases the activity of matrix metalloproteinase 1 and collagenases produced by monocytes and macrophages, which contributes to the destabilization of atherosclerotic plaque [35]. CRP also displays thrombogenic activity via potentiation of thromboxane activity [36]. Another study demonstrated a positive correlation between the intensity of staining for the presence of CRP in atherosclerotic lesions in coronary arteries, the concentration of this protein in plasma, and the number of unstable plaques with a thin fibrous cap [37]. Forte et al. showed that in patients with ACS, CRP is produced and released within the coronary circulation, which is associated with impairment of endothelial function [38]. 

## 5. Limitations of the Study

Several limitations to our study should be acknowledged. First, findings of our study due to its case-control design associated with the potential for confounding may be rather hypothesis-generating than definitive. Second, our cases and controls slightly differed in terms of age and gender distribution. However, the strength of the association between CRP concentration and the ACS occurrence in our study was even greater after adjustment for these variables. Third, we enrolled a broad spectrum of ACS patients and detailed estimation of the relationship between CRP concentration and the ACS occurrence may vary among different ACS types. However, our inclusion criteria reflect a real-world setting and at the early stage of ACS, when we collected blood samples, it is usually unlikely to differentiate between NSTEMI and UA. Fourth, we cannot exclude the modulatory effect of prior statin therapy given in a substantial proportion of cases on the obtained results. Fifth, we accounted in our calculations neither for diurnal and seasonal variations in CRP concentration nor for physical activity of the study participants. Sixth, in our study we evaluated exclusively CRP, apolipoproteins, and the traditional lipid profile. It remains unclear whether novel biomarkers such as high-sensitivity cardiac troponins, myeloperoxidase, growth differentiation factor-15, and interleukin 1 receptor-like 1 (ST2) possess an additional predictive value to that obtained from CRP measurement and validated risk scores [39, 40]. 

## 6. Conclusions

Our study indicates that CRP superiorly to apolipoproteins and lipid profile facilitates the risk stratification for ACS occurrence. However, large prospective cohort trials are required to verify our findings and assess whether novel biomarkers possess an additional predictive value to that obtained from CRP measurement and validated risk scores.

## Figures and Tables

**Table 1:** Baseline characteristics of the study participants.

Parameter	ACS patients (*n* = 220)	Control group (*n* = 116)	*P *
Age (years)	64 ± 12	52 ± 9	0.01
TC (mmol/L)	4.96 (4.11–5.99)	4.75 (4.26–5.09)	0.024
LDL-C (mmol/L)	3.18 (2.35–3.93)	2.79 (2.3–3.15)	0.002
HDL-C (mmol/L)	1.16 ± 0.31	1.45 ± 0.31	<0.0001
Non-HDL-C (mmol/L)	3.85 (3.1–4.65)	3.2 (2.79–3.54)	<0.0001
TG (mmol/L)	1.3 (0.99–1.89)	0.92 (0.71–1.88)	<0.0001
TC : HDL-C	4.43 (3.72–5.42)	3.23 (2.82–3.72)	<0.0001
TG : HDL-C	2.77 (1.88–4.08)	1.47 (0.99–2.08)	<0.0001
LDL-C : HDL-C	2.77 (2.16–3.55)	1.85 (1.56–2.27)	<0.0001
apoA-I (g/L)	1.27 ± 0.24	1.41 ± 0.31	<0.0001
apoB (g/L)	0.81 (0.65–1.00)	0.72 (0.61–0.86)	0.0009
apoB : apoA-I	0.64 (0.52–0.8)	0.51 (0.45–0.62)	<0.0001
hsCRP (mg/L)	2.79 (1.12–6.08)	0.69 (0.36–1.35)	<0.0001
BMI (kg/m^2^)	26.8 (24.5–29.8)	24.4 (22.1–27.7)	0.014
Women	41% (91)	53% (61)	<0.05
Men	59% (129)	47% (55)	
Dyslipidemia	87% (191)	57% (66)	<0.00001
Hypertension	74% (163)	20% (23)	<0.00001
Diabetes mellitus	26% (57)	0	<0.00001
Smoking	54% (119)	27% (31)	0.0001
Family history of premature CAD	35% (77)	26% (30)	ns
Prior statin use	69% (152)	5% (6)	<0.0001

ACS: acute coronary syndrome; apoA-I: apolipoprotein A-I; apoB: apolipoprotein B; apoB : apoA-I: apolipoprotein B to apolipoprotein A-I ratio; BMI: body mass index; CAD: coronary artery disease; HDL-C: high-density lipoprotein cholesterol; hsCRP: high-sensitivity C-reactive protein; LDL-C: low-density lipoprotein cholesterol; LDL-C : HDL-C: low-density lipoprotein cholesterol to high-density lipoprotein cholesterol ratio; non-HDL-C: nonhigh-density lipoprotein cholesterol; TC: total cholesterol; TC : HDL-C: total cholesterol to high-density lipoprotein cholesterol ratio; TG: triglycerides; TG : HDL-C: triglycerides to high-density lipoprotein cholesterol ratio.

**Table 2:** Characteristics of patients according to the type of ACS.

Parameter	UA (*n* = 96)	NSTEMI (*n* = 57)	STEMI (*n* = 67)	*P* value	Post hoc*
Age (years)	63 ± 10	64 ± 12	66 ± 14	ns	—
TC (mmol/L)	4.83 (3.93–5.99)	5.27 (4.52–6.33)	4.8 (4.11–5.76)	ns	—
LDL-C (mmol/L)	2.84 (2.3–3.77)	3.69 (2.99–4.11)	3.18 (2.45–3.8)	0.007	UA versus STEMI
HDL-C (mmol/L)	1.13 ± 0.33	1.89 ± 0.31	1.16 ± 0.28	ns	—
Non-HDL-C (mmol/L)	3.74 (2.97–4.55)	4.27 (3.62–5.17)	3.67 (2.89–4.39)	0.022	UA versus STEMI
TG (mmol/L)	1.37 (1.01–2.08)	1.2 (0.85–1.6)	1.28 (0.98–1.97)	ns	—
TC : HDL-C	4.28 (3.73–5.2)	4.52 (3.78–5.45)	4.43 (3.30–5.31)	ns	—
TG : HDL-C	2.89 (2.05–4.71)	2.32 (1.7–3.63)	3.03 (1.96–4.02)	ns	—
LDL-C : HDL-C	2.5 (1.98–3.25)	3.2 (2.5–4.03)	2.76 (1.93–3.36)	0.005	UA versus STEMI
apoA-I (g/L)	1.27 ± 0.27	1.29 ± 0.22	1.26 ± 0.23	ns	—
apoB (g/L)	0.76 (0.59–0.95)	0.88 (0.79–1.1)	0.78 (0.64–0.92)	0.001	UA versus STEMI
					STEMI versus NSTEMI
apoB : apoA-I	0.61 (0.47–0.77)	0.73 (0.6–0.89)	0.63 (0.48–0.76)	0.005	UA versus STEMI
					STEMI versus NSTEMI
hsCRP (mg/L)	2.13 (0.96–4.63)	3.58 (1.49–6.86)	3.6 (1.4–9.07)	0.008	UA versus STEMI
					UA versus NSTEMI
BMI (kg/m^2^)	26.7 (24.7–31.2)	26.2 (24.1–29.3)	27.4 (24.6–28.9)	ns	—

*Presence of a statistically significant difference in the post hoc analysis.

apoA-I: apolipoprotein A-I; apoB: apolipoprotein B; apoB : apoA-I: apolipoprotein B to apolipoprotein A-I ratio; BMI: body mass index; HDL-C: high-density lipoprotein cholesterol; hsCRP: high-sensitivity C-reactive protein; LDL-C: low-density lipoprotein cholesterol; LDL-C : HDL-C: low-density lipoprotein cholesterol to high-density lipoprotein cholesterol ratio; non-HDL-C: nonhigh-density lipoprotein cholesterol; ns: not significant; NSTEMI: non-ST elevation myocardial infarction; STEMI: ST elevation myocardial infarction; TC: total cholesterol; TC : HDL-C: total cholesterol to high-density lipoprotein cholesterol ratio; TG: triglycerides; TG : HDL-C: triglycerides to high-density lipoprotein cholesterol ratio; UA: unstable angina.

**Table 3:** Effect of conventional risk factors on the probability of ACS development.

Variable	Risk estimation		
	OR	95% CI	*P *
Age (for a 10-year increase)	5.49	3.36–8.97	<0.00001
Diabetes mellitus	—	—	—
Dyslipidemia	4.98	2.92–8.53	<0.00001
Family history of premature CAD	1.38	0.76–2.52	ns
Hypertension	12.9	6.7–24.9	<0.00001
Sex (male versus female)	1.28	0.81–2.00	ns
Smoking	3.22	1.78–5.81	0.0001

ACS: acute coronary syndrome; CAD: coronary artery disease; CI: confidence interval; ns: not significant; OR: odds ratio.

**Table 4:** The risk of the acute coronary syndrome for primary variables.

Parameter	Crude odds ratio	Adjusted odds ratio		
	Model 0* OR (95% CI) *P* value for the model	Model 1 OR (95% CI) *P* value for the model	Model 2 OR (95% CI) *P* value for the model	Model 3 OR (95% CI) *P* value for the model
TC	1.08 (0.94–1.22)	1.10 (0.98–1.23)	1.12 (1.01–1.25)	1.04 (0.83–1.3)
	ns	ns	0.029	ns
LDL-C	0.94 (0.82–1.07)	0.92 (0.82–1.03)	0.9 (0.81–1.00)	0.99 (0.8–1.23)
	ns	ns	0.05	ns
HDL-C	0.89 (0.78–1.01)	0.9 (0.8–1.00)	0.88 (0.8–0.98)	0.84 (0.67–1.06)
	ns	ns	0.019	ns
TG	0.99 (0.96–1.02)	0.99 (0.97–1.02)	0.98 (0.96–1.01)	1.00 (0.96–1.05)
	ns	ns	ns	ns
apoA-I	0.99 (0.98–1.01)	0.96 (0.94–0.99)	0.97 (0.95–0.99)	0.99 (0.96–1.02)
	ns	0.002	0.005	ns
apoB	1.01 (0.99–1.03)	1.01 (0.98–1.03)	1.01 (0.98–1.04)	1.01 (0.95–1.07)
	ns	ns	ns	ns
hsCRP	1.60 (1.32–1.94)	1.55 (1.21–1.98)	1.64 (1.26–2.12)	1.66 (1.2–2.29)
	0.0001	0.0001	0.0001	0.002

*Model 0 unadjusted: *χ* ^2^ = 131.7; df = 7; *P* < 0.001; log-likelihood = 275.5; *R* ^2^ Cox and Snell = 0.34; *R* ^2^ Nagelkerke = 0.47.

Model 1 adjusted for age: *χ* ^2^ = 241.9; df = 8; *P* < 0.001; log-likelihood = 165.3; *R* ^2^ Cox and Snell = 0.53; *R* ^2^ Nagelkerke = 0.74.

Model 2 adjusted for age and sex: *χ* ^2^ = 251.0; df = 9; *P* < 0.001; log-likelihood = 156.2; *R* ^2^ Cox and Snell = 0.54; *R* ^2^ Nagelkerke = 0.75.

Model 3 adjusted for age, sex, and smoking status: *χ* ^2^ = 219.7; df = 10; *P* < 0.001; log-likelihood = 64.6; *R* ^2^ Cox and Snell = 0.62; *R* ^2^ Nagelkerke = 0.86.

apoA-I: apolipoprotein A-I; apoB: apolipoprotein B; CI: confidence interval; HDL-C: high-density lipoprotein cholesterol; hsCRP: high-sensitivity C-reactive protein; LDL-C: low-density lipoprotein cholesterol; ns: not significant; OR: odds ratio; TC: total cholesterol; TG: triglycerides.

**Table 5:** The risk of the acute coronary syndrome for primary and secondary variables.

Parameter	Crude odds ratio	Adjusted odds ratio		
	Model 0* OR *P* value for the model	Model 1 OR (95% CI) *P* value for the model	Model 2 OR (95% CI) *P* value for the model	Model 3 OR (95% CI) *P* value for the model
TC	1.10 (0.95–1.29)	1.08 (0.96–1.22)	1.11 (0.98–1.26)	1.59 (0.66–3.83)
	ns	ns	ns	ns
LDL-C	0.90 (0.78–1.04)	0.87 (0.78–0.98)	0.85 (0.75–0.95)	0.59 (0.24–1.45)
	ns	0.024	0.007	ns
HDL-C	0.88 (0.75–1.04)	0.95 (0.82–1.1)	0.92 (0.80–1.07)	0.42 (0.15–1.16)
	ns	ns	ns	ns
TG	0.99 (0.95–1.02)	1.01 (0.97–1.04)	0.99 (0.96–1.03)	0.80 (0.63–1.01)
	ns	ns	ns	ns
apoA-I	0.99 (0.96–1.02)	0.96 (0.92–1.01)	0.96 (0.92–1.01)	1.02 (0.87–1.2)
	ns	ns	ns	ns
apoB	0.99 (0.94–1.05)	0.99 (0.92–1.06)	0.99 (0.92–1.07)	0.97 (0.75–1.26)
	ns	ns	ns	ns
hsCRP	1.60 (1.31–1.95)	1.56 (1.21–2.01)	1.64 (1.26–2.13)	1.90 (1.34–2.89)
	0.0001	0.001	0.0001	0.001
nonHDL-C	1.01 (0.97–1.04)	1.04 (0.99–1.09)	1.05 (0.99–1.1)	1.32 (0.85–2.04)
	ns	ns	ns	ns
TC : HDL-C	0.76 (0.8–6.81)	6.42 (0.32–125.6)	6.01 (0.25–145.3)	0.00 (0.27–104797)
	ns	ns	ns	ns
TG : HDL-C	0.92 (0.37–2.3)	0.36 (0.13–0.99)	0.38 (0.13–1.08)	976 (0.17–5713045)
	ns	0.05	ns	ns
LDL-C : HDL-C	2.75 (0.49–15.37)	0.92 (0.11–7.83)	0.72 (0.73–7.03)	12.1 (0.00–1.878*E*1)
	ns	ns	ns	ns
apoB : apoA-I	0.50 (0.001–402.2)	1.09 (0.01–10916)	0.39 (0.000–1853)	18.2 (0.000–4.117*E*1)
	ns	ns	ns	ns

*Model 0 unadjusted: χ^2^ = 139.2; df = 12; *P* < 0.001; log-likelihood = 268.0; *R* ^2^ Cox and Snell = 0.35; *R* ^2^ Nagelkerke = 0.49.

Model 1 adjusted for age: χ^2^ = 251.8; df = 13; *P* = 0.001; log-likelihood = 155.4; *R* ^2^ Cox and Snell = 0.54; *R* ^2^ Nagelkerke = 0.75.

Model 2 adjusted for age and sex: χ^2^ = 259.6; df = 14; *P* < 0.001; log-likelihood = 147.6; *R* ^2^ Cox and Snell = 0.55; *R* ^2^ Nagelkerke = 0.77.

Model 3 adjusted for age, sex, and smoking status: χ^2^ = 233.4; df = 15; *P* < 0.001; log-likelihood = 50.9; *R* ^2^ Cox and Snell = 0.64; *R* ^2^ Nagelkerke = 0.90.

apoA-I: apolipoprotein A-I; apoB: apolipoprotein B; apoB : apoA-I: apolipoprotein B to apolipoprotein A-I ratio; CI: confidence interval; HDL-C: high-density lipoprotein cholesterol; hsCRP: high-sensitivity C-reactive protein; LDL-C: low-density lipoprotein cholesterol; LDL-C : HDL-C: low-density lipoprotein cholesterol to high-density lipoprotein cholesterol ratio; non-HDL-C: nonhigh-density lipoprotein cholesterol; ns: not significant; OR: odds ratio; TC: total cholesterol; TC : HDL-C: total cholesterol to high-density lipoprotein cholesterol ratio; TG: triglycerides; TG : HDL-C: triglycerides to high-density lipoprotein cholesterol ratio.

**Table 6:** Diagnostic usefulness of the assayed parameters for the occurrence of ACS.

Variable	Cut-off value	Sensitivity	Specificity	Positive predictive value	Negative predictive value
CRP (mg/L)	0.85*	0.84	0.62	86%	58%
	3.00^#^	0.48	0.89	90%	46%
TC : HDL-C	3.72	0.77	0.79	88%	63%
LDL-C : HDL-C	2.15	0.76	0.70	84%	59%
TG : HDL-C	1.55	0.85	0.59	81%	66%
apoB : apoA-I	0.53	0.74	0.56	77%	51%

*The optimal cut-off value for hsCRP test determined by ROC analysis in our study.

^#^A lower limit for the high risk category according the CDC and AHA statement on markers of inflammation and cardiovascular disease [6].

ACS: acute coronary syndrome; AHA: the American Heart Association; apoB : apoA-I: apolipoprotein B to apolipoprotein A-I ratio; CDC: the Centers for Disease Control and Prevention; CRP: C-reactive protein; LDL-C : HDL-C: low-density lipoprotein cholesterol to high-density lipoprotein cholesterol ratio; TC : HDL-C: total cholesterol to high-density lipoprotein cholesterol ratio; TG : HDL-C: triglycerides to high-density lipoprotein cholesterol ratio.
